# Peritoneal Metastasis After Treated With Abemaciclib Plus Fulvestrant for Metastatic Invasive Lobular Breast Cancer: A Case Report and Review of the Literature

**DOI:** 10.3389/fendo.2021.659537

**Published:** 2021-10-08

**Authors:** Hong-Fei Gao, Jun-Sheng Zhang, Qiang-Zu Zhang, Teng Zhu, Ci-Qiu Yang, Liu-Lu Zhang, Mei Yang, Fei Ji, Jie-Qing Li, Min-Yi Cheng, Gang Niu, Kun Wang

**Affiliations:** ^1^ Department of Breast Cancer, Cancer Center, Guangdong Provincial People’s Hospital, Guangdong Academy of Medical Sciences, Guangzhou, China; ^2^ Shantou University Medical College, Shantou, China; ^3^ Phil Rivers Technology, Beijing, China

**Keywords:** breast, lobular carcinoma, neoplasm metastasis, peritoneum, whole exome sequencing

## Abstract

Peritoneal metastases from invasive lobular carcinoma (ILC) of breast are uncommon and usually related to poor prognosis due to difficulty of detection in clinical practice and drug resistance. Therefore, recognizing the entities of peritoneal metastases of ILC and the potential mechanism of drug resistance is of great significance for early detection and providing accurate management. We herein report a case of a 60-year-old female who presented with nausea and vomiting as the first manifestation after treated with abemaciclib (a CDK4/6 inhibitor) plus fulvestrant for 23 months due to bone metastasis of ILC. Exploratory laparotomy found multiple nodules in the peritoneum and omentum, and immunohistochemistry confirmed that the peritoneal metastatic lesions were consistent with ILC. Palliative therapy was initiated, but the patient died two months later due to disease progression with malignant ascites. Whole exome sequencing (WES) was used to detect the tumor samples and showed the peritoneal metastatic lesions had acquired ESR1 and PI3KCA mutations, potentially explaining the mechanism of endocrine therapy resistance. We argue that early diagnosis of peritoneal metastasis from breast cancer is crucial for prompt and adequate treatment and WES might be an effective supplementary technique for detection of potential gene mutations and providing accurate treatment for metastatic breast cancer patients.

## Introduction

Invasive breast cancer is a histologically diverse disease that has several defined histological subtypes. Invasive breast carcinoma of no special type (IBC-NST), which presents in 70%-75% of the cases, is the most common histologic subtype of breast cancer, followed by invasive lobular carcinoma (ILC), which accounts for only 5%-15% of invasive mammary carcinomas (1, 2). ILC was more likely estrogen receptor positive, HER-2 negative and had a lower proliferative index compared to IBC-NST (3).

ILC, with the hallmark loss of E-cadherin expression, is characterized by its infiltrating growth behavior, which invades the surrounding tissue with a single-file pattern at histologic examination (3, 4). Compared with IBC-NST, ILC displays a predilection for distant metastasis to uncommon sites such as gastrointestinal (GI) tract, peritoneum and genitourinary system (5, 6), and has slightly worse prognosis (7). Peritoneal metastases of breast cancer are challenging for clinicians to diagnose promptly, and recognition of the entities is of great significance for early detection and providing accurate management. As for HR-positive metastatic breast cancer (MBC), hormonal therapy represents the backbone of treatment. Recently, a new class of molecular drug, cyclin-dependent kinase 4/6 (CDK4/6) inhibitors, has been proved to improve efficacy of the first- or second-line treatment of HR positive, HER2-negative MBC (8–13). Herein, we present a rare case of metastatic ILC with peritoneal metastases causing bowel obstruction during the treatment with abemaciclib (a CDK4/6 inhibitor) plus fulvestrant.

## Case Presentation

A 60-year-old female with no family history of cancer underwent left mastectomy with axillary lymph node dissection in November 2012 for stage IIIC (cT2N3M0) invasive lobular carcinoma. Histopathological examination demonstrated an invasive lobular carcinoma with positive estrogen receptor (ER+) and progesterone receptor (PgR+), negative human epidermal growth factor receptor 2 (HER2-), lymphovascular invasion and metastases to axillary lymph nodes (11/21). The pathological stage was pT2N3M0. She completed 4 cycles of epirubicin and cyclophosphamide (EC) followed by 4 cycles of paclitaxel. Then the patient underwent adjuvant radiotherapy; specifically, the left chest wall, infraclavicular and supraclavicular region, and internal mammary nodes were irradiated at a dose of 50.4 Gy in 5 weeks with a 1.8 Gy daily fraction. Meanwhile, she received once-daily regimen of letrozole 2.5 mg regularly. Follow-up was arranged every 3 months for 2 years in the breast clinic and there was no distinct evidence of recurrence. Then, the patient was followed up every 6 months in the next 3 years.

In December 2017 (5 years after surgery), bone scan detected solitary bone metastasis in the left ischium. She received intravenous zoledronic acid injections every month. She was subjected to abemaciclib plus fulvestrant with stable disease until her current presentation.

**Figure 1:**
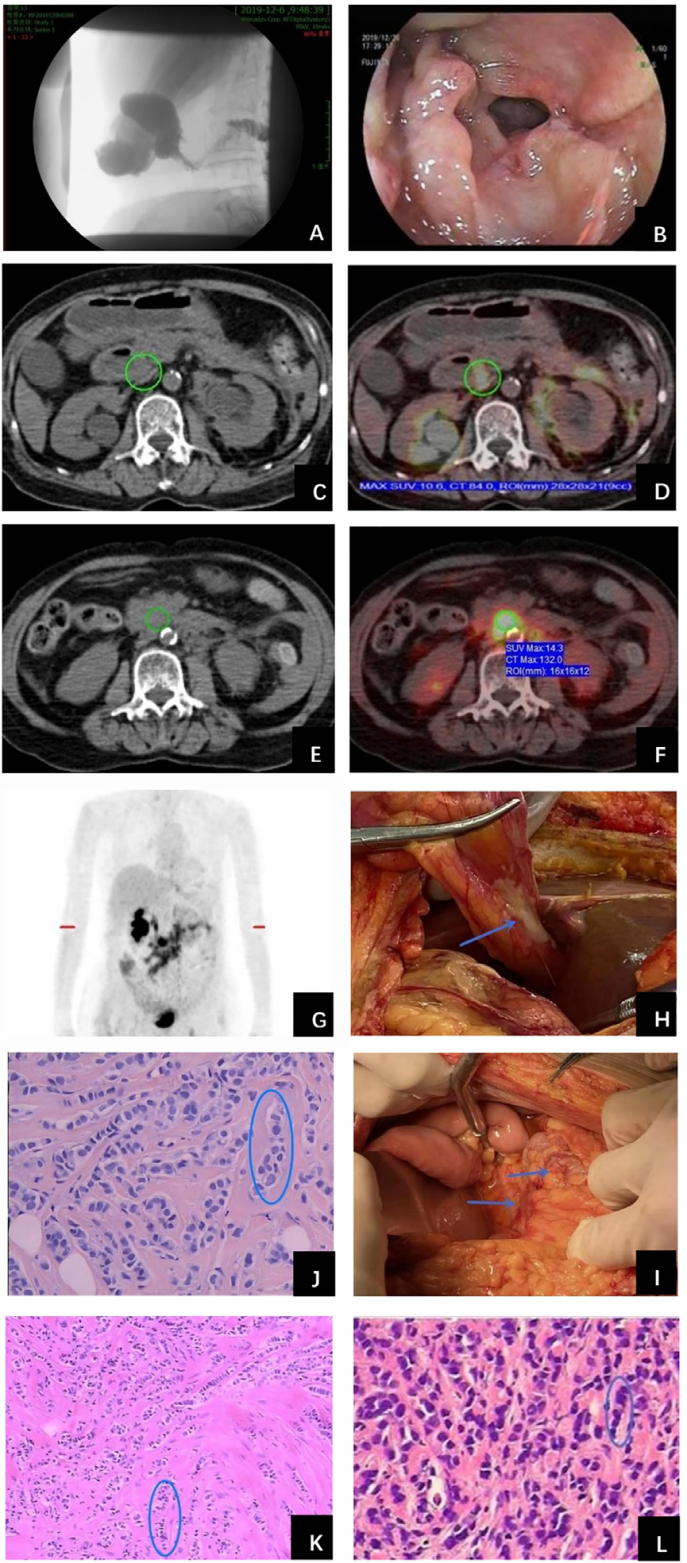
Examinations and histopathological results during work-up of the patient. **(A)** Upper gastrointestinal X-ray showed a stricture in the second portion of the duodenum. **(B)** Upper gastrointestinal endoscopy detected a stricture with circumferential edematous friable mucosa, extending from the duodenal bulb to the second portion of the duodenum. **(C)** PET/CT revealed duodenal wall was thickened and identified as metabolically active lesions (SUVmax=10.6) **(D)**. **(E)** Thickened peritoneum and mesenteries and slightly larger lymph nodes in the mesenteries were found with intense FDG uptake (SUVmax=14.3) **(F)**. **(G)** Holistic view of PET/CT: metabolic lesions in the duodenum, peritoneum and mesenteries. Exploratory laparotomy showed three metastatic nodules in the peritoneal cavity, including one nodule on the ligamentum teres hepatis **(H)** and the other two on the omentum **(I)** (arrows). Histopathological examination of primary breast cancer **(J)**, metastatic axillary lymph node **(K)** and metastatic peritoneal nodule **(L)** all revealed single-file strands of infiltrating small tumor cells dispersed in the fibrous matrix (circle).

**Figure 2:**
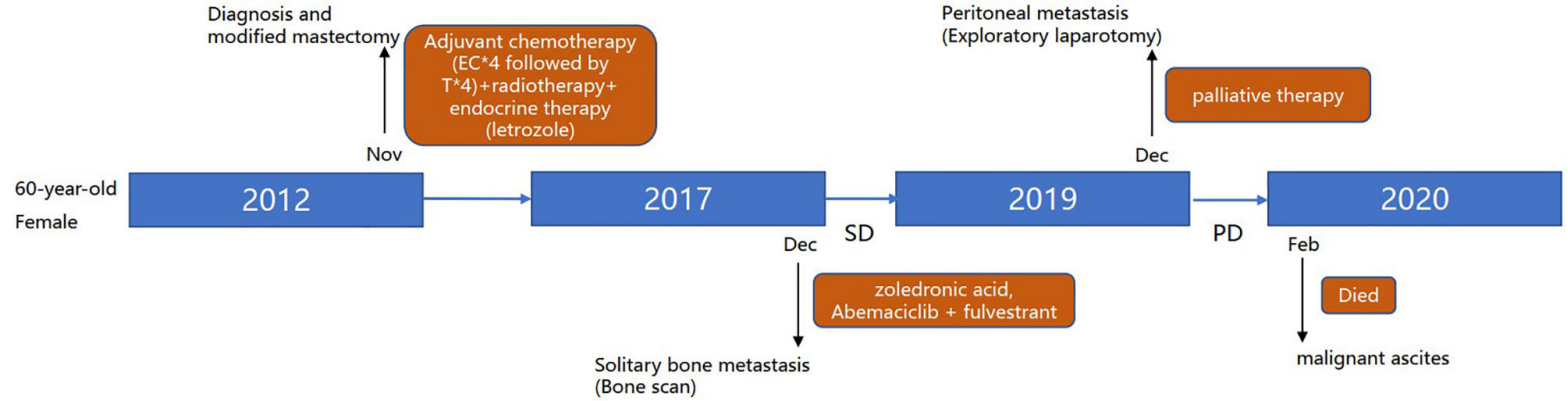
Review of treatment process of the patient (from November 2012 to February 2020).

In November 2019, the patient complained of nausea. A contrasted abdominal Computed Tomography (CT) showed no distinct abnormalities. The patient took some prescribed medication and felt better thereafter. However, vomiting after meals occurred in December 2019. The patient came to our hospital for further treatment. She presented with jaundice and mild tenderness in the upper abdomen on admission. Blood chemistry tests showed elevated bilirubin and liver enzymes. The laboratory workup showed CA153 186.8U/ml, CA199 203.5U/ml, CA125 66.27U/ml, and CEA 13.2U/ml. An upper gastrointestinal X-ray (**Figure 1A**) and upper gastrointestinal endoscopy (**Figure 1B**) showed a stricture in the horizontal part of duodenum which had poor distension. A biopsy obtained from the duodenum did not detect any malignant cell. Positron Emission Tomography/Computed Tomography (PET/CT) showed thickening of duodenal wall, peritoneum and mesenteries, slightly larger lymph nodes in the mesenteric area, and varying degrees of increase in glucose metabolism (**Figures 1C−G**); combined with perirenal, duodenal and bladder lesions, peritonitis was highly suspected, while tuberculous peritonitis was supposed to be excluded. Thereafter, a decision was made to perform an exploratory laparotomy. In the operation, approximately 1 liter of yellow-brown ascitic fluid was drained and three nodules were seen in the peritoneal cavity, including one nodule on the ligamentum teres hepatis (**Figure 1H**) and the other two on the omentum (**Figure 1I**). Similar to the primary breast cancer (**Figure 1J**) and metastatic axially lymph node (**Figure 1K**), histological examination of the peritoneal nodule showed single-file strands of infiltrating tumor cells throughout the fibrous matrix, which were consistent with ILC (**Figure 1L**). The immunohistochemical (IHC) studies revealed the tumor cells were highly positive for gross cystic disease fluid protein-15 (GCDFP-15), Cytokeratin 7 (CK7), GATA-3 and ER, but negative for E-cadherin, PR and HER2 status; and the Ki67 index was 20%. The results of IHC staining were consistent with a diagnosis of peritoneal metastases from ILC. During the process of diagnosis, the patient manifested with severer nausea and vomiting and even abdominal distention, and she was received parenteral nutrition instead of oral feeding. Due to the poor condition of the patient, aggressive treatment such as chemotherapy was not considered for her, and finally palliative therapy was initiated. Unfortunately, the patient died two months later due to disease progression with malignant ascites (**Figure 2**).

**Figure 3:**
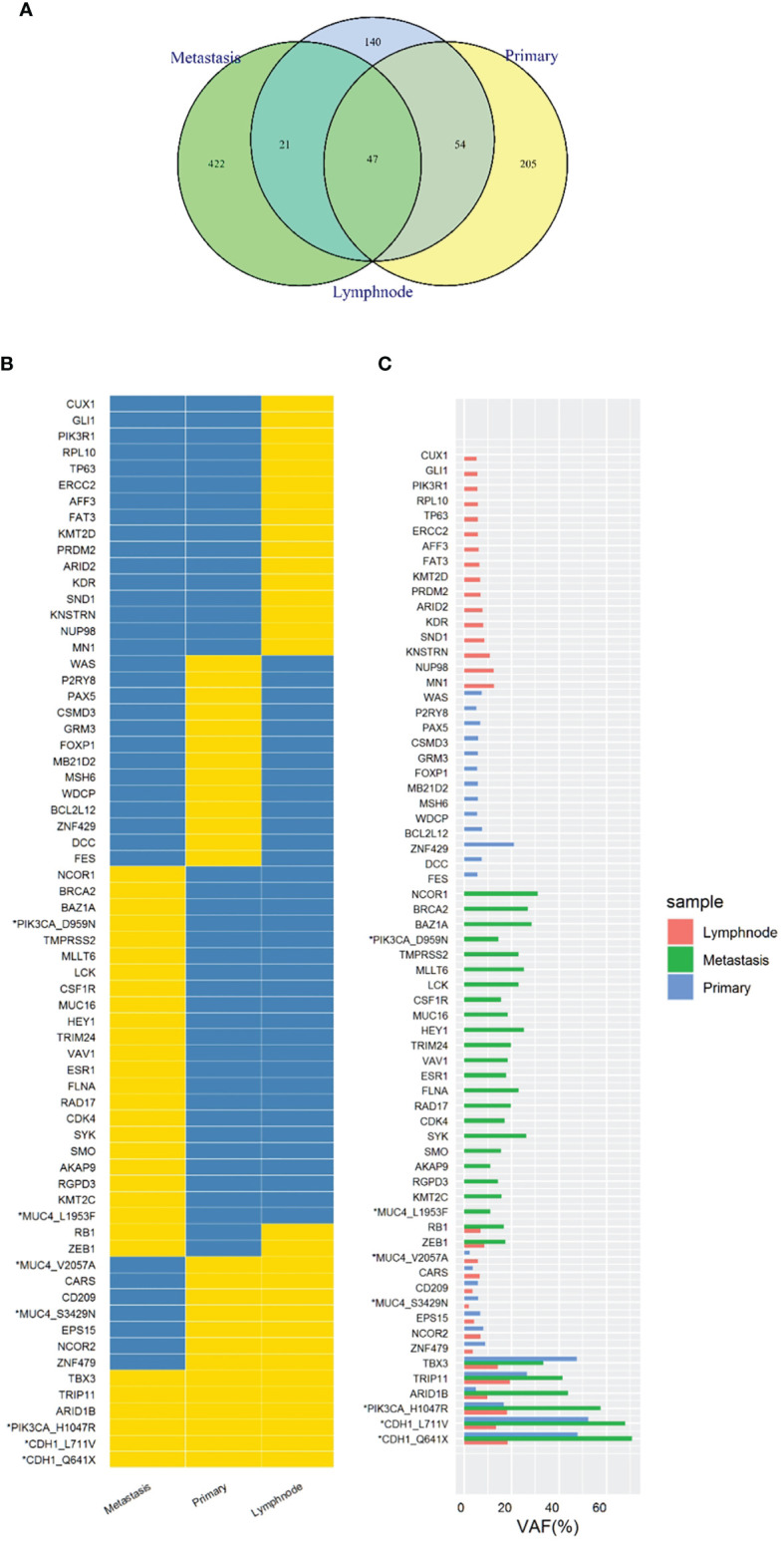
Whole exome sequencing (WES) of tumor samples from primary lesion, regional lymph nodes and peritoneal metastatic lesions. **(A)** 3-way Venn Diagram showed the mutational overlaps in the three samples. There were 47 common mutations in the three samples, while another 21 common mutations between lymph node and metastatic site, and another 54 common mutations between lymph node and primary site. **(B)** Somatic mutation heatmap. The mark “*” means that there are 2 or more mutations in the same gene, which was labelled with gene or amino acid changes. Yellow means there is variation, while blue means there is no variation. **(C)** Variation frequency (VAF) distribution. The mark “*” means that there are 2 or more mutations in the same gene, which was labelled with gene or amino acid changes.

**Figure 4:**
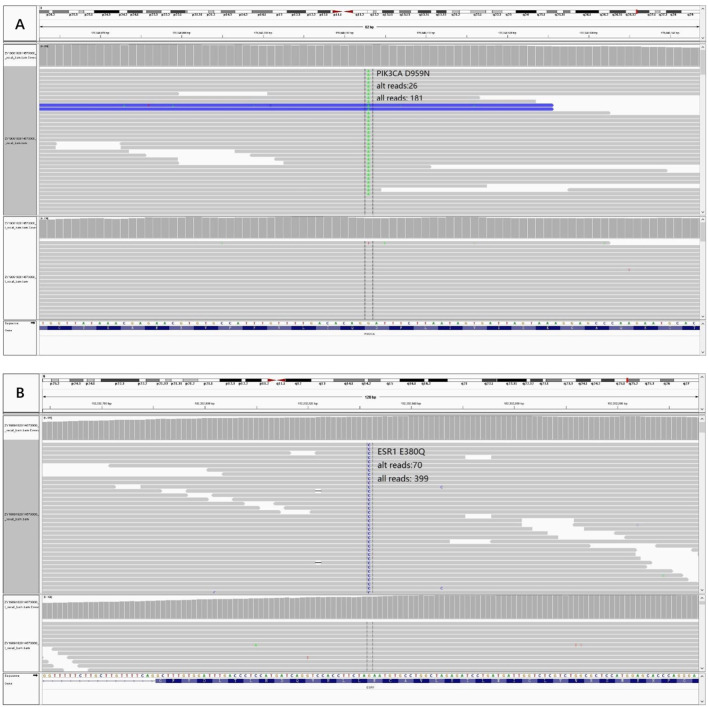
Acquired mutations were detected in the sample of peritoneal metastatic lesion and visualized through Integrative Genomics Viewer (IGV). **(A)** Variant PIK3CA p.D959N IGV plot (all reads: 181, alternative allele supported reads: 26). **(B)** Variant ESR1 p.E380Q IGV plot (all reads: 399, alternative allele supported reads: 70).

In order to know the patient’s genetic information and investigate the possible mechanisms of resistance to endocrine therapy (ET), we utilized whole exome sequencing (WES) to detect the tumor samples from primary lesion, regional lymph nodes and peritoneal metastatic lesions. The 3-way Venn Diagram showed that 47 common mutations were detected among primary lesion, lymph nodes and peritoneal metastatic lesions (**Figure 3A**); combined with somatic mutation heatmap (**Figure 3B**) and variation frequency (VAF) distribution (**Figure 3C**), it was implied that the three tumor samples may have the same origin. Mutation analysis of signal transduction enrichment showed that the PI3K-AKT signaling pathway was significantly enriched in the peritoneal metastatic lesions. All three tumor samples carried PIK3CA p.H1047R, which is one of the most common mutation of PIK3CA in breast cancer. What’ more, the sample of metastatic lesion was found to have acquired PIK3CA p.D959N (**Figure 4A**) and ESR1 p.E380Q mutation (**Figure 4B**), which were not detected in neither primary lesion nor lymph node.

## Discussion

The patient in this case was found to have peritoneal metastasis from ILC after diagnostic work-up for the presence of nausea and vomiting. Peritoneal carcinomatosis secondary to breast cancer has been reported in literatures (**Table 1**) (14–21), and some studies showed nearly 3% of ILC patients had peritoneal metastasis, which was higher than those of IBC-NST patients (22, 23).

**Table 1:** Review of literature: characteristics and outcomes of breast cancer patients with peritoneal metastasis.

First Authors	Age(years)	TNMstage	Molecular Type	Surgery	Adjuvant Therapies	DFS^*^	Clinical Characteristics of Metastasis	Metastasis Site	Diagnosis and treatment	Outcomes
J.A. Mosiun (14)	51	T2N2M0	ER(-), PR(-), and equivocal for HER2	Mastectomy and axillary dissection	3*FEC-3*taxotere; radiotherapy	2 years	Have a right iliac fossa mass on abdominal examination.	Terminal ileum wall, multiple peritoneal nodules and enlarged intraabdominal lymph nodes	Right hemicolectomy with creation of double barrel stoma; oral letrozole and intravenous zoledronic acid injections	Disease progression with malignant ascites
Yasuhiro Nihon-yanagi (15)	57	T2N1M0	ER(+), PgR(+), HER2(-)	Modified radical mastectomy with axillary and infraclavicular lymph-node dissection	8*paclitaxel; tamoxifen	17 months	Nausea, vomiting and jaundice	Duodenum, Peritoneum	Pancreatoduodenectomy	Bilateral hydronephrosis; subsequently died
R. Syed (16)	63	T2N0M0	ER(+), PgR(-)	Mastectomy with axillary node sampling	Local radiotherapy; tamoxifen	5 years	abdominal pain, distension and diarrhea	Omentum, peritoneum and pelvis	Ascitic drainage, tissue biopsy of an omental deposit	Unknown
Kobayashi T (17)	56	T4bN1M0	ER(+)	Modified radical mastectomy	Unknown	Unknown	Abdominal distension, vomiting and epigastric pain	Peritoneum and retroperitoneum	Gastrojejunostomy; cisplatin, 5-fluorouracil, doxifluridine, and TS1	Unknown
Foluso O. Ademuyiwa (18)	81	Unknown	Unknown	Mastectomy	Unknown	41 years	Fatigue, nausea, vomiting, weight loss, and a right lower quadrant abdominal mass	Left periaortic soft tissue, right intraabdominal soft tissue, falciform ligament and bilateral perinephric fat	CT-guided biopsy of the periaortic mass; weekly paclitaxel and zoledronic acid	Unknown
Osaku (19)	69	/	/	/	/	/	Constipation	Abdominal cavity, rectum and ileocecum	Exploratory laparotomy; hormone therapy and taxane- and anthracycline-based drugs	Died four years later
I. Mylonas (20)	76	Unknown	Unknown	Mastectomy and axillary lymphadenectomy	No	30 years	loss of appetite, nausea, vomiting and abdominal enlargement without weight gain	Greater epiploon, peritoneum, ileum and uterus	Biopsies of the greater epiploon	Unknown
Aurello (21)	73	T2N1M0	ER(+), PgR(+)	Mastectomy with axillary node dissection	Chemotherapy	14 years	Vomiting, epigastric pain and weight loss	Angulus, Peritoneum	Subtotal gastrectomy with D1-lymphadenectomy and stapled gastrojejununi anastomosis, chemotherapy	Free from disease until March 2004 when she revealed a peritoneal carcinosis

^*^ DFS, disease-free survival.

Clinical manifestations of peritoneal metastasis from ILC are variable and non-specific. Patients usually do not have any symptoms until later, even several days before death. Metastasis to the peritoneum or retroperitoneum leads to thickening and sclerosis of the surrounding tissues. A common finding of peritoneum metastasis from breast cancer is stenosis, frequently with presentation of abdominal pain, early satiety and obstructive symptoms. These patients might be misdiagnosed with a primary GI tumor or even not diagnosed with malignancy at all (24, 25). A case series of 12, 001 patients found 11% of the patients were not diagnosed with GI metastasis from breast carcinoma until an exploratory laparotomy was performed, as was the patient in our case (6). As the clinical presentation of peritoneal metastases is usually non-specific, histopathological and immunohistochemical examinations are the definitive diagnostic methods. Microscopically, single-file strands of infiltrating tumor cells invading the surrounding tissue can frequently be seen in metastases from ILC as observed in our study (26, 27). However, it is still quite challenging to come up with the definite diagnosis *via* histological examination because signet ring cell carcinoma can indeed arise from any tissue. Immunohistochemical markers are crucial for diagnosing metastatic lobular carcinoma of the breast. The most important markers for ILC are Cytokeratin 7 (CK7), GATA-3, gross cystic disease fluid protein-15 (GCDFP-15), ER and PR, all of which but not PR were highly positive in the biopsy specimens of peritoneal metastatic lesions in our patient (28, 29). There was also negative of HER2. Samples from distant sites often show features similar to that of primary breast cancer which is most commonly an ILC. The availability of IHC studies allowed clinicians to accurately diagnose metastatic lobular breast carcinoma (30, 31).

There is no consensus for the treatment of peritoneal carcinomatosis secondary to breast cancer, as there have not yet been any large-scale studies that compared the efficacy of different managements (30, 31). Palliative surgery is necessary in the treatment of patients with symptomatic obstruction, bleeding or perforation, even though no survival benefit may ensue (24, 30, 32). A few studies have been published describing the combination of surgical debulking and hyperthermic intraperitoneal chemotherapy (HIPEC) for patients with secondary peritoneal carcinomatosis due to breast cancer as well as other primary diseases, which showed improvement in morbidity and mortality (33, 34). A retrospective study (6), which included 73 breast cancer patients with GI or peritoneal metastasis, reported palliative surgical intervention conferred no survival benefit while systemic chemotherapy or hormone therapy might have improved survival of the patients. Late presentation of signs and symptoms of peritoneal metastasis was related to poor prognosis. However, there is no enough data in the best treatment and precise prognosis for those patients due to the limited number of case reports. Treatment should be tailored to the patient and their projected performance status along with quality-of-life consideration (35, 36).

In this case, ILC metastasized to the peritoneum and omentum in association with spread of many small nodules. It remains unclear concerning the mechanism of these metastatic patterns. Previous study (37) revealed that most of ILC lack cohesiveness because the E-cadherin was inactivated, which was a cell-to-cell adhesion protein. WES showed the patient had gene mutations in ESR1 and PIK3CA at the metastatic lesions, which was thought to be acquired due to chronic exposure of CDK4/6 inhibitor plus ET (38). As the most common mechanism of resistance to ET in MBC, acquired ESR1 mutations may have been existing in primary tumors and become enriched only when metastasis occurs (39). By enhancing coactivator recruitment, ESR1 mutations with altered structure conferred distinct mechanism of resistance to ER antagonists such as tamoxifen (40, 41). Previous studies demonstrated that MBC patients with ESR1 mutation are resistant to standard ET and have worse overall survival (42, 43), as seen in our patient. Currently, the best treatment for MBC patients with ESR1 mutations is fulvestrant combined with CDK4/6 inhibitor, which conferred significantly improved PFS in patients with ESR1 mutations (39). In this case, the patient was treated with abemaciclib plus fulvestrant after solitary bone metastasis, and the PFS was 23 months. Besides to ESR1 mutation, the PIK3CA mutation was also found in the metastatic lesions. Some studies (44, 45) demonstrated that the PI3K/mTOR pathway was upregulated in response to long-term use of CDK4/6 inhibitor, which drove cell cycle progression *via* upregulating cyclin D. Therefore, PIK3CA mutation and the subsequently activated PI3K-AKT signaling pathway might mediate resistance to CDK4/6 inhibitor for this patient. The SOLAR-1 trial (46) showed patients with PIK3CA mutation had double PFS after receiving PIK3CA inhibitor alpelisib plus fulvestrant compared with those receiving fulvestrant plus placebo (11.0 months and 5.7 months, respectively). In the subgroup of patients who had been treated with CDK4/6 inhibitors previously, receiving alpelisib reduced 52% risk in PFS compared with placebo (47). Hence, PIK3CA inhibitors may be used to overcome resistance to CDK4/6 inhibitor for MBC patients. However, PIK3CA inhibitors including alpelisib are not available in the mainland of China up to now. Meanwhile, ILC patients with peritoneal metastasis usually progress quickly and are easily misdiagnosed, which makes it difficult for these patients to acquire timely and effective treatment. Therefore, it is crucial that more efforts need to be put into early detection of ILC patients with peritoneal metastasis and availability of new drugs like PIK3CA inhibitors.

By using WES to detect the tumor samples, it is available for us to get access to the genomic information of this patient and investigate the possible mechanism of endocrine therapy resistance. WES showed the peritoneal metastatic lesions had acquired ESR1 and PI3KCA mutations, potentially explaining the mechanism of endocrine therapy resistance. Therefore, we argue that early diagnosis of peritoneal metastasis from breast cancer is crucial for prompt and adequate treatment and WES might be an effective supplementary technique for detection of potential gene mutations and providing accurate treatment for metastatic breast cancer patients.

## Conclusion

All clinicians should realize that there is an unusual pattern of peritoneal metastasis from ILC. For patients with vomiting and previous history of ILC, it is necessary to highly suspect peritoneum metastasis. Early diagnosis is vital in ensuring prompt and adequate treatment. Our results suggest that ESR1 and PIK3CA mutations are acquired resistance mechanism of CDK4/6 inhibitor plus endocrine therapy and WES might be an effective supplementary technique for detection of potential gene mutations for MBC patients with drug resistance, thus ensuring timely and accurate salvage treatment. Nevertheless, further studies need to be conducted to investigate the mechanism and predictive factors of peritoneal metastasis of ILC.

## Data Availability Statement

The original contributions presented in the study are included in the article. Further inquiries can be directed to the corresponding authors.

## Ethics Statement

Written informed consent was obtained from the individual(s) for the publication of any potentially identifiable images or data included in this article.

## Author Contributions

H-FG and J-SZ was mainly responsible for the article writing. Q-ZZ, and FJ was mainly responsible for the gene analysis. KW and GN were in charge of all study procedures. TZ, C-QY, L-LZ and MY were responsible for patient’s clinical data and analysis. J-QL and M-YC were responsible for consent from the patient and ethics committee. All authors gave final approval of the manuscript to be submitted and agreed to be accountable for all aspects of the work.

## Funding

This study is supported by grants from National Natural Science Foundation of China (82171898, 82103093), Science and Technology Planning Project of Guangzhou City (202002030236), Beijing Medical Award Foundation (YXJL-2020-0941-0758), Guangdong Basic and Applied Basic Research Foundation (2020A1515010346, 2021A1515011570), Guangzhou Science and Technology Project (202102021055), Fundamental Research Funds for the Central Universities (2020ZYGXZR017), Science and Technology Special Fund of Guangdong Provincial People’s Hospital (2017zh01), CSCO-Hengrui Cancer Research Fund (Y-HR2016-067), and Guangdong Provincial Department of Education Characteristic Innovation Project (2015KTSCX080). Funding sources were not involved in the study design, data collection, analysis and interpretation, writing of the report, or decision to submit the article for publication.

## Conflict of Interest

Author GN and Q-ZZ was employed by the company Phil Rivers Technology, China.

The remaining authors declare that the research was conducted in the absence of any commercial or financial relationships that could be construed as a potential conflict of interest.

## Publisher’s Note

All claims expressed in this article are solely those of the authors and do not necessarily represent those of their affiliated organizations, or those of the publisher, the editors and the reviewers. Any product that may be evaluated in this article, or claim that may be made by its manufacturer, is not guaranteed or endorsed by the publisher.

## Supplementary Material

The Supplementary Material for this article can be found online at: https://www.frontiersin.org/articles/10.3389/fendo.2021.659537/full#supplementary-material
